# Supplementary material for: Populus simonii × Populus nigra overexpressing PsnWRKY70 recruits phyllosphere bacterial strains that inhibit Alternaria alternata
Source: mSystems. 2025 Aug 15;10(9):e01765-24. doi: 10.1128/msystems.01765-24 (PMC12455959; doi:10.1128/msystems.01765-24)
Supplement: Supplemental Figures — Figures S1 to S6. [file msystems.01765-24-s0001.docx]

**SUPPLEMENTARY INFORMATION**

***Populus simonii × P***. ***nigra* overexpressing** ***PsnWRKY70* recruits phyllosphere bacterial strains that inhibit** ***Alternaria alternata***

Wei Wang^a1,4,5^, Weixiong Wang^a1,2,3^, Jing Jiang^1,3^, Xiangdong Bai^1,7^, Kun Chen^1,6^, Xiaoyue Zhang^1,6^, Jingya Yang^1,2,3^, Di Wu^1,2,3^*, Ben Niu^1,2,3^*, Guifeng Liu^1,3^*.

1 State Key Laboratory of Tree Genetics and Breeding, Northeast Forestry University, Harbin 150040, China

2 College of Life Science, Northeast Forestry University, Harbin 150040, China

3 The Center for Basic Forestry Research, Northeast Forestry University, Harbin 150040, China

4 Peking University Institute of Advanced Agricultural Sciences, Shandong Laboratory of Advanced Agriculture Sciences in Weifang, Weifang, Shandong, 261325, China

5 College of Life Sciences, Shandong Agricultural University, Taian, Shandong, 271018, China

6 School of Life Sciences, Qilu Normal University, Jinan, 250200, China

7 State Key Laboratory of Plant Cell and Chromosome Engineering, Institute of Genetics and Developmental Biology, Innovation Academy for Seed Design, Chinese Academy of Sciences, Beijing 100101, China

*Authors for correspondence:

*Di Wu*

*Email: wudi_nefu@nefu.edu.cn*

*Ben Niu*

*Email: ben_niu@nefu.edu.cn*

*Guifeng Liu*

*Email:* [*liuguifeng@126.com*](mailto:liuguifeng@126.com)

^a^ These authors contributed equally

**SUPPLEMENTARY RESULTS**

**
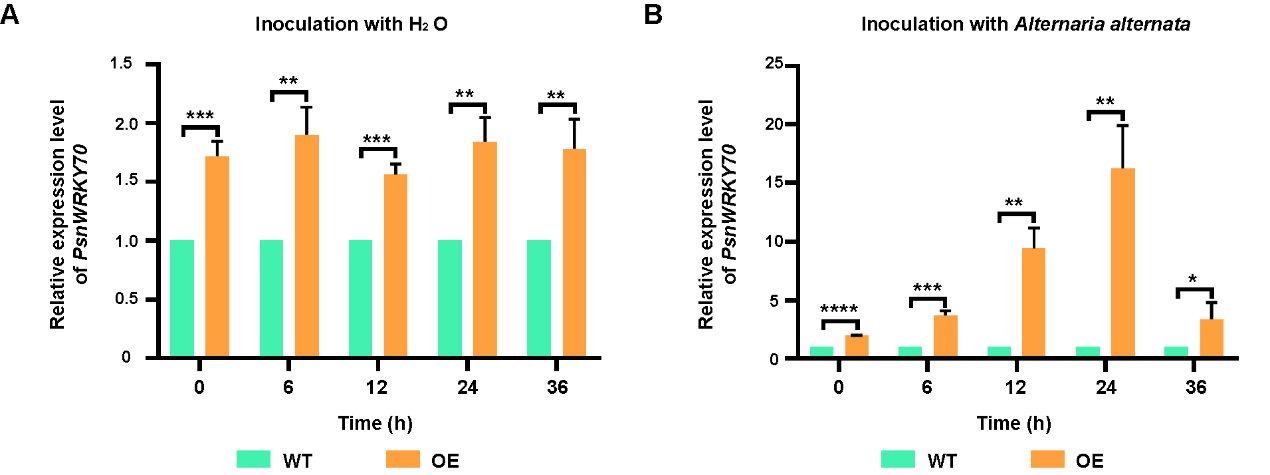
**

**Figure S1** **Expression levels of the *PsnWRKY70* gene in the WT and OE lines of *Populus* measured after inoculation with H_2_O (A) and *Alternaria alternata* (B) over a period of 0–36 h.**


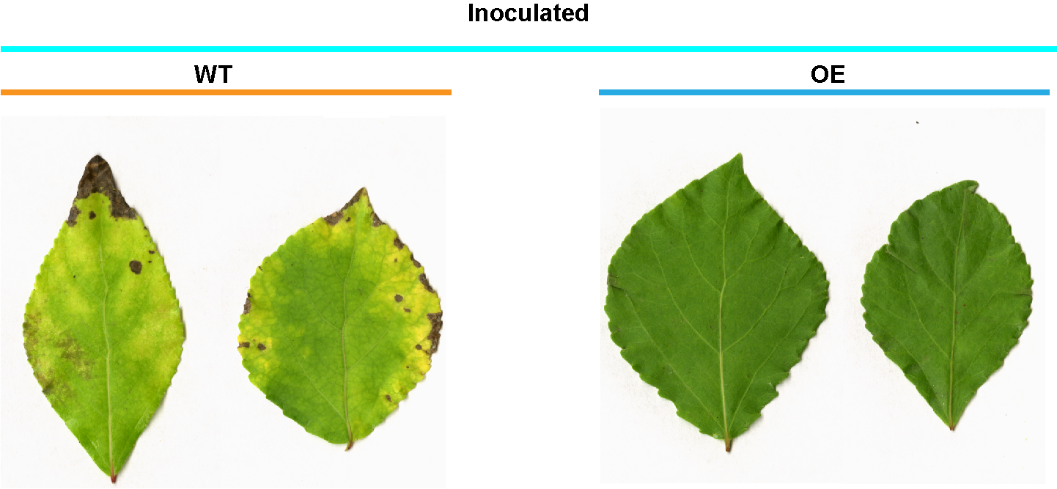


**Figure S2 Symptoms on the leaves of WT and OE lines of *Populus* inoculated with *Alternaria alternata*.**


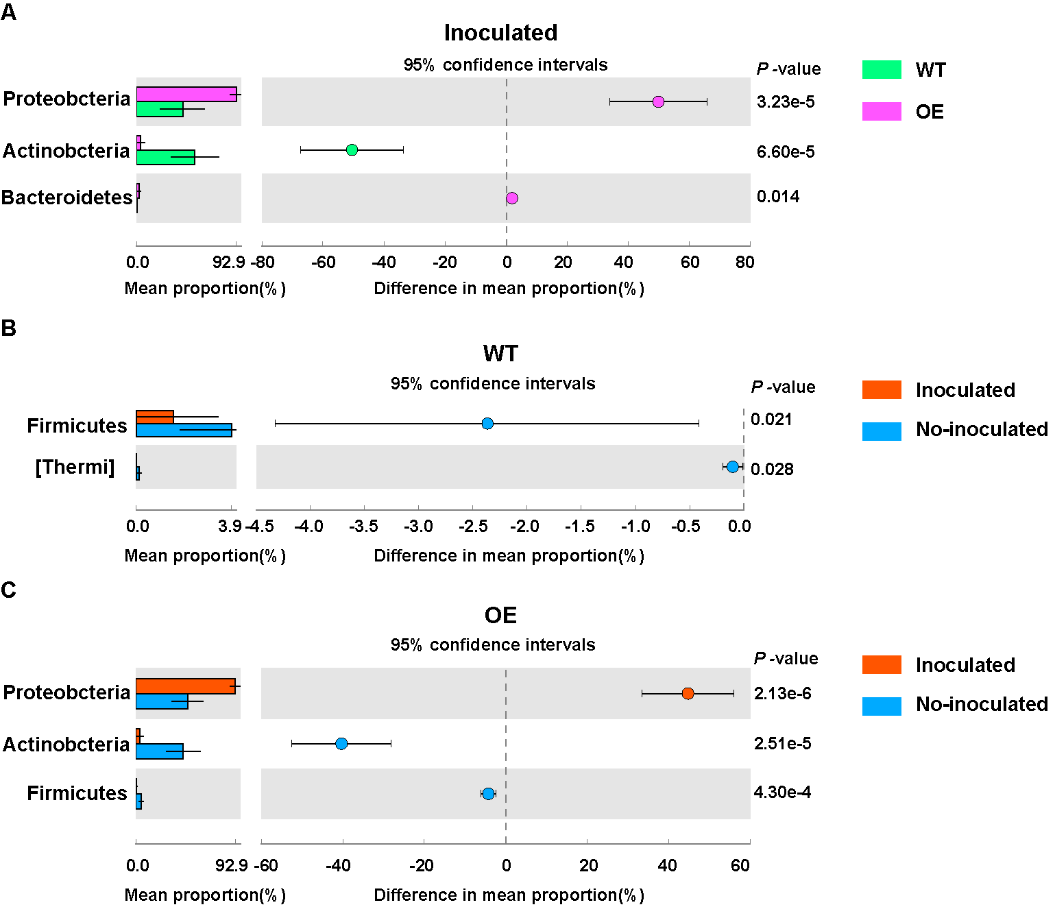


**Figure S3** **Comparison of the relative abundance of the dominant phyla in phyllosphere microbial communities between the OE and WT lines of *Populus* inoculated with *Alternaria alternata* (A)** **Comparison of the relative abundance of the dominant phyla in the WT (B) or OE (C) lines that were non-inoculated and inoculated with *A*. *alternata*.**

Signiﬁcant differences at the phyla level were analyzed using a two-sided Welch’s t-test at a *P*-value of 0.05.


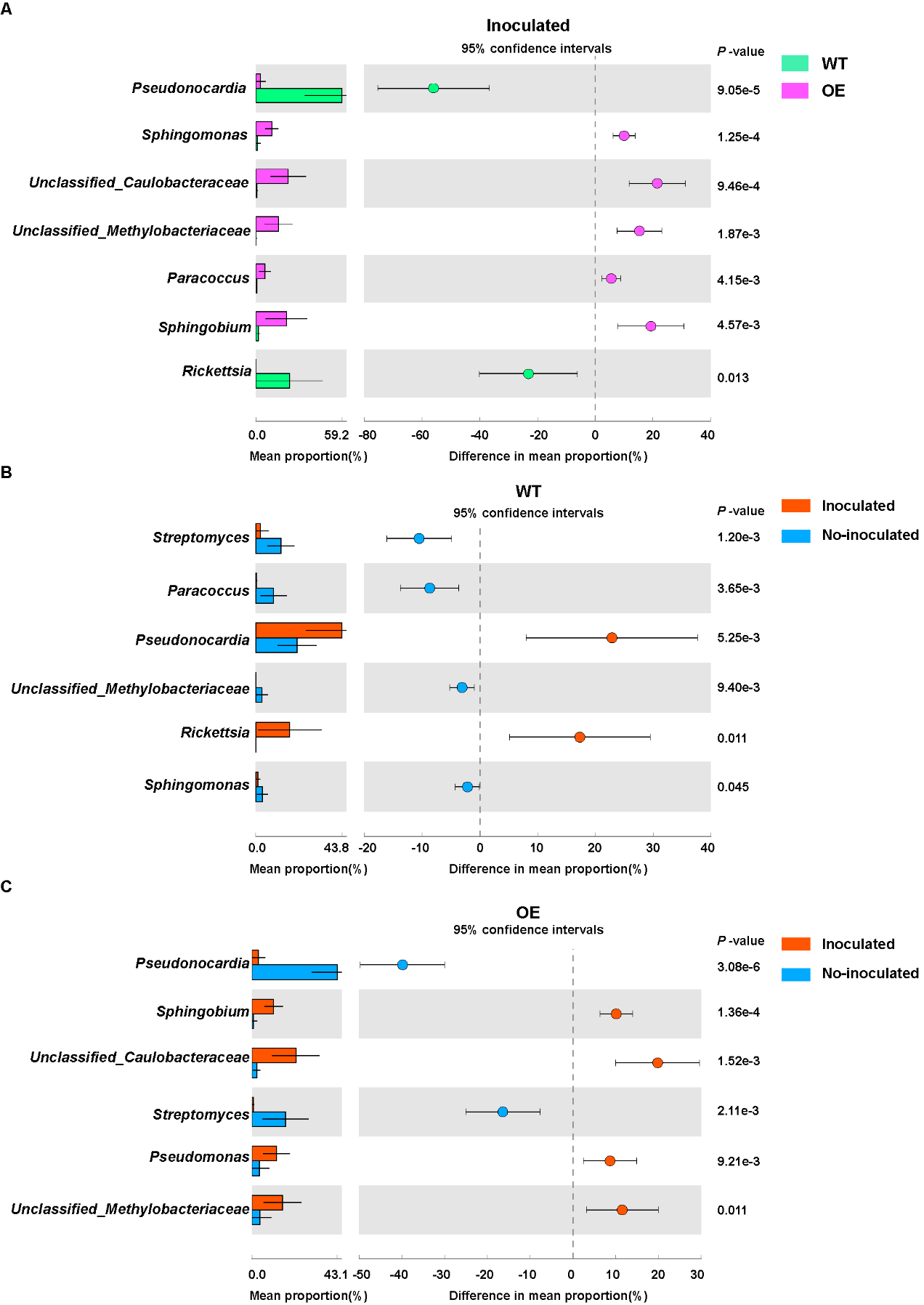


**Figure S4 Comparison of the relative abundance of the dominant genera in phyllosphere microbial communities between the OE and WT lines of *Populus* inoculated with *Alternaria alternata.* (A)** **Comparison of the relative abundance of the dominant genera in the WT (B) or OE (C) lines that were non-inoculated and inoculated with *A*. *alternata*.**

Signiﬁcant differences at genera level were analyzed using a two-sided Welch’s t-test at a *P*-value of 0.05.

**
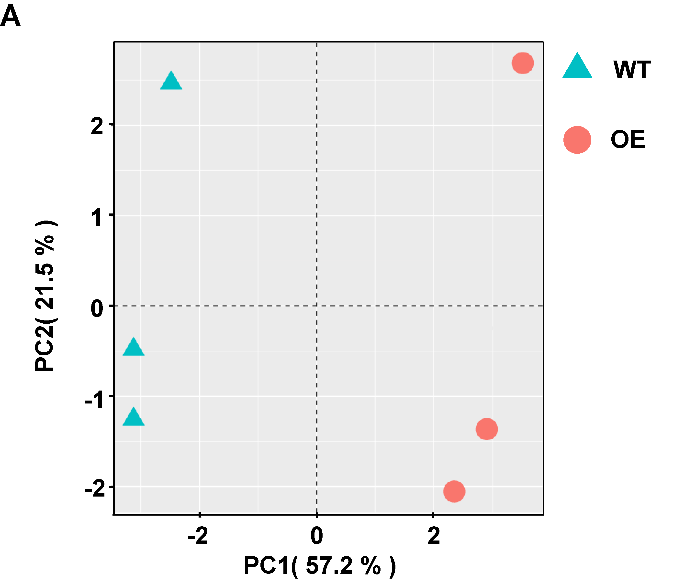
**

**Figure S5 Principal component analysis of phyllosphere metabolites from the WT and OE lines of *Populus* inoculated with *A. alternata*.**

**
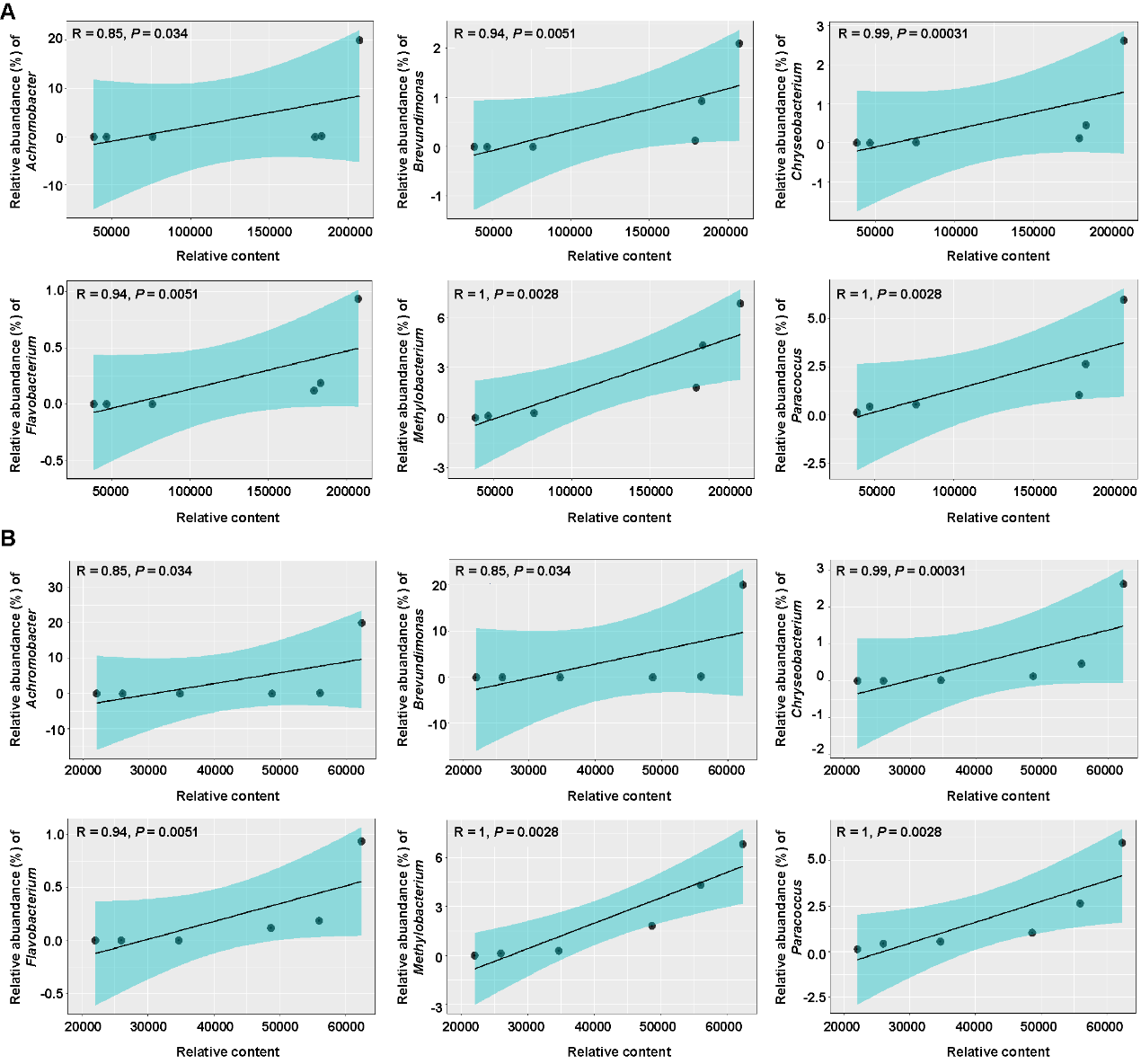
**

**Figure S6 Correlation between the relative abundance of the genera from the six biocontrol bacteria isolates and the contents of fraxin (A) and scopolin (B) in the leaves of the WT and OE lines inoculated with *A. alternata*. Shading represents the 95% conﬁdence interval.**
